# Two‐Dimensional Heterostructure Complementary Logic Enabled by Optical Writing

**DOI:** 10.1002/smsc.202300319

**Published:** 2024-03-01

**Authors:** Ayaz Ali, Matthias Schrade, Wen Xing, Per Erik Vullum, Ozhan Koybasi, Takashi Taniguchi, Kenji Watanabe, Branson D. Belle

**Affiliations:** ^1^ Department of Smart Sensor Systems SINTEF DIGITAL Forskningsveien 1 Oslo 0373 Norway; ^2^ Research Center for Frontier Fundamental Studies Zhejiang Lab Hangzhou 311100 China; ^3^ Department of Electronic Engineering University of Sindh Jamshoro 76080 Pakistan; ^4^ Department of Sustainable Energy Technology SINTEF Forskningsveien 1 Oslo 0373 Norway; ^5^ Department of Materials and Nanotechnology SINTEF Høgskoleringen 5 Trondheim 7034 Norway; ^6^ Department of Microsystems and Nanotechnology (MiNaLab) SINTEF Digital Oslo 0373 Norway; ^7^ Research Center for Materials Nanoarchitectonics National Institute for Materials Science 1‐1 Namiki Tsukuba 305‐0044 Japan; ^8^ Research Center for Electronic and Optical Materials National Institute for Materials Science 1‐1 Namiki Tsukuba 305‐0044 Japan

**Keywords:** 2D materials, complementary metal‐oxide semiconductor, field effect transistors, logic inverters

## Abstract

Integrated logic circuits using atomically thin, two‐dimensional (2D) materials offer several potential advantages compared to established silicon technologies such as increased transistor density, circuit complexity, and lower energy dissipation leading to scaling benefits. In this article, a novel approach to achieve tunable doping in 2D semiconductors is explored to achieve complementary transistors and logic integration. By selectively transferring WSe_2_ onto hBN and SiO_2_ substrates, complementary transistor behavior (n‐ and p‐type) was achieved using a UV light source and electrostatic activation. Furthermore, advanced characterization techniques, including high‐resolution transmission electron microscopy (HRTEM) and Kelvin probe force microscopy (KPFM), provided insights into the chemical composition and surface potential changes after UV writing. Finally, a logic inverter was successfully implemented using selectively photo‐induced doped WSe_2_ transistors, showcasing the potential for practical logic applications. This innovative method opens new avenues for designing energy‐efficient and reconfigurable 2D semiconductor circuits, addressing key challenges in modern electronics.

## Graphical abstract

We show complementary logic using a single flake of few layer WSe_2_ which has been transferred half on hBN and SiO_2_ substrates. Exposure of the flake to UV illumination with an applied negative gate voltage, manipulates the charge carrier type creating p‐ and n‐type transistors. HRTEM shows the effect of the writing process, and a proof‐of‐concept logic inverter is demonstrated.

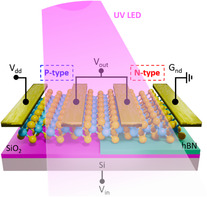

## 1 Introduction

The standard complementary metal‐oxide semiconductor (CMOS) implementation of logic circuits uses both p‐ and n‐type field effect transistors (FETs), fabricated on the same silicon chip.^[^ ^1^ ^]^ Compared to earlier technologies consisting of only either n‐ or p‐type FETs, the main advantage of CMOS logic is higher energy efficiencies as energy dissipation mostly occurs during switching events, although continuous downscaling has increased the contribution of static energy dissipation also in complementary technologies. As a result, heat dissipation is a major problem for modern processors and the size of cooling units has increased in line with the advances in computing power.^[^ ^2^ ^]^ It is therefore clear that technologies beyond silicon must be complementary, i.e., based on both p‐ and n‐type FETs, to keep heat dissipation to a minimum.

Two‐dimensional (2D) semiconductors such as transition metal dichalcogenides (TMDCs) have garnered significant interest due to their remarkable properties which include high carrier mobility, trap‐free surfaces without dangling bonds, high in‐plane thermal conductivity for efficient heat dissipation, and band gap tunability.^[^ ^3^, ^4^, ^5^, ^6^ ^]^ These material properties allow the realization of FETs with excellent performance metrics for logic applications such as on/off current ratios of up to 10^8^ and subthreshold swing of down to 60 mV dec^−1^.^[^ ^7^ ^]^ Several groups have recently reported the fabrication and characterization of initially simple but increasingly complex logic circuits in 2D materials.^[^ ^8^, ^9^, ^10^, ^11^, ^12^, ^13^, ^14^, ^15^, ^16^, ^17^ ^]^ However, most of this work is based on only one type of carrier type, for example, n‐type in the case of the most studied material MoS_2_, and the benefits of a complementary implementation in 2D materials remain largely unexplored. The difficulty in achieving the required conduction type in 2D materials relates to the problem that doping strategies from established bulk semiconductor technologies, namely silicon, cannot be simply transferred to these materials. In silicon, p‐ and n‐type regions are defined by implantation of high energy ions. This strategy would lead to beam damage and device degradation if applied to devices made from atomically thin materials.^[^ ^18^ ^]^ Complementary logic circuits have thus been realised using intrinsic p‐ and n‐type 2D materials normally on separate chips, for example, MoS_2_ and WSe_2_ for, respectively, the n‐ and p‐type FET's.^[^ ^19^ ^]^ However, due to complicated fabrication processes, scalability concerns, and integration challenges, alternative concepts to control carrier type and concentration within the same channel material are needed.

Charge transfer doping from adsorbed species^[^ ^20^, ^21^, ^22^ ^]^ or deposited materials,^[^ ^23^, ^24^ ^]^ channel thickness or interfacial layer thickness modulation^[^ ^25^, ^26^ ^]^ and metal contacts with different work functions^[^ ^24^, ^27^ ^]^ are a few of the strategies that have been employed in an effort to influence the carrier type of 2D semiconductors. However, these techniques cause unpredictable charge transfer and Fermi level pinning at intrinsic defects, resulting in weak tunability of the transport properties of these devices^[^ ^21^, ^26^ ^]^ in addition to significant charge carrier scattering.^[^ ^27^ ^]^ Another approach for complementary integration of 2D materials uses electrostatic doping via so called polarity gates, which turns different regions of the device into p‐ and n‐type conductors when biased.^[^ ^28^ ^]^ This approach offers the additional flexibility of reconfigurability by simply changing the applied voltage but increases the device footprint and fabrication complexity due to additional contacts and added deposited layers.

A recently emerging strategy towards reconfigurable control of transport properties is optical doping.^[^ ^29^ ^]^ For example, Seo et al.^[^ ^30^ ^]^ recently demonstrated an innovative method based on light illumination to achieve reconfigurable doping in 2D materials. In this case, a single flake of MoTe_2_ on SiO_2_ substrate was sequentially illuminated with two light sources of different wavelengths (532 and 355 nm). The illuminated areas of the flake then exhibited n‐ and p‐ type behaviour resulting in complementary logic.

In this article, we use a single light source to flood illuminate a single sheet of WSe_2_ which was selectively transferred on hBN and SiO_2_ substrates. Although a similar strategy has been used to dope MoTe_2_ flakes,^[^ ^31^ ^]^ they did not achieve ambipolarity of their doped devices. In our case, we achieved controllable p‐ and n‐type polarity leading to an ambipolar homojunction resulting in a demonstrable logic inverter logic. We further show that this doping strategy leads to the diffusion of B and N atoms to the surface of the WSe_2_ layer, where hBN is the substrate contributing to changes in the Fermi level of the WSe_2_ conducting layer. Moreover, we suggest, that the substrate can be selectively patterned to increase the transistor density and circuit complexity leading to scaling benefits.

## 2 Results and Discussions

### 2.1 Device Structure and Transport Properties

**Figure 1:**
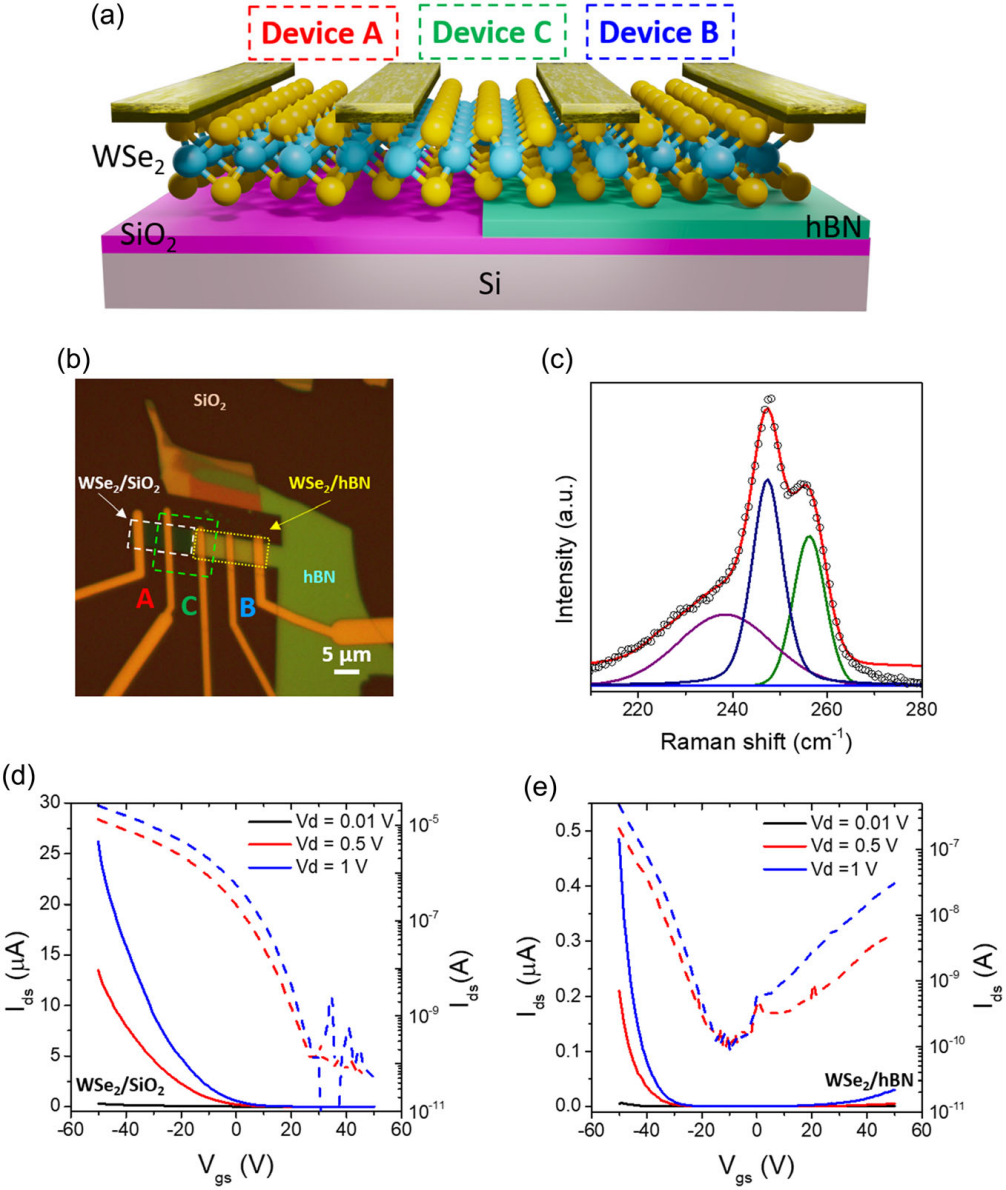
Device structure and transport properties. a) Schematic of WSe_2_/SiO_2_ (Device A) and WSe_2_/hBN/SiO_2_ (Device B) FET devices, b) Optical image of a fabricated device, c) Raman spectrum of WSe_2_ on SiO_2_ substrate, d,e) Transfer characteristics of the WSe_2_/SiO_2_ and WSe_2_/hBN/SiO_2_ FET native devices under different drain‐source voltages both in linear (solid line) and log scale (dashed line).

The structural arrangement of the WSe_2_ FETs employed in our study is presented in **Figure** **1**a in which a WSe_2_ flake is directly transferred on the SiO_2_/Si substrate and a hBN flake using a dry transfer technique^[^ ^32^ ^]^ such that half the flake spans the hBN and SiO_2_. This type of device structure provides an excellent system to directly compare the transport properties of WSe_2_/SiO_2_ (Device A) and WSe_2_/hBN/SiO_2_ (Device B) FET devices. Figure 1b shows an optical microscopy image of the WSe_2_/SiO_2_ and WSe_2_/hBN/SiO_2_ FET devices. For clarity, we emphasize that the same WSe_2_ flake is used to create both the SiO_2_ and hBN/SiO_2_ devices. Raman spectrum of WSe_2_ on SiO_2_ is presented in Figure 1c. The E^1^_2g_ band at 247.5 cm^−1^ and A_1g_ band at 256.5 cm^−1^ which correspond to the in‐plane and out‐of‐plane vibrations suggest that the WSe_2_ is trilayer.^[^ ^33^ ^]^ The number of WSe_2_ layers was also confirmed by high‐resolution transmission electron microscopy (HRTEM) (Figure S3, Supporting Information). Raman spectroscopy of WSe_2_/hBN/SiO_2_ was also conducted and compared with WSe_2_/SiO_2_, see supporting information Figure S2, Supporting Information. There is a slight relative red shift in the peak positions of 0.7 cm^−1^ which corresponds to p‐type doping of WSe_2_ on hBN.^[^ ^21^ ^]^ The electrical characterization of the fabricated devices was carried out in dark conditions. Figure 1d,e show the transfer characteristics of the WSe_2_/SiO_2_ and WSe_2_/hBN/SiO_2_ FETs, respectively. For effective comparison between both devices, the channel length was kept identical (5 μm). The gate‐source bias (*V*_gs_) was scanned from +50 to −50 V and the drain‐source current (*I*_ds_) was recorded under different drain‐source voltages (0.01, 0.5, and 1 V). Device A exhibits unipolar conduction (p‐type) with a maximum p‐*I*_ON_ current of 27 μA at *V*_gs_ –50 V and *V*_ds_ 1 V. The device has a hole mobility of *μ*_h_ = 45.69 cm^−2^ V^−1^s^−1^ and the minimum conduction point (MCP) was located beyond +50 V while device B shows ambipolar transport with a dominant p‐branch having a maximum p‐*I*_ON_ current of 0.5 μA at the same gate voltage, hole mobility of *μ*_h_ = 5.10 cm^−2^ V^−1^s^−1^ and the MCP shifted to −10 V. The corresponding output curves are shown in Figure S4, Supporting Information where it is observed that device A exhibits near ohmic behavior whereas device B shows non‐ohmic behavior for the same metal contacts (Ti/Au) deposited at the same time under the same conditions. This points to a difference in the work function of the WSe_2_ layer on hBN, which leads to increased contact resistance and less drain‐source current. The absence of ambipolarity in device A could be due to the Fermi level pinning at the thin WSe_2_/SiO_2_ interface where charge trapping occurs in oxide defect bands.^[^ ^34^ ^]^ In contrast, hBN is well known as an ideal substrate for 2D materials and forms ultraclean interfaces with fewer charge trapping centers.^[^ ^35^, ^36^, ^37^ ^]^ This leads us to infer that hBN reduces Fermi level pinning thereby enabling the manifestation of ambipolar transport in device B.

### 2.2 Effect of Optical Writing

**Figure 2:**
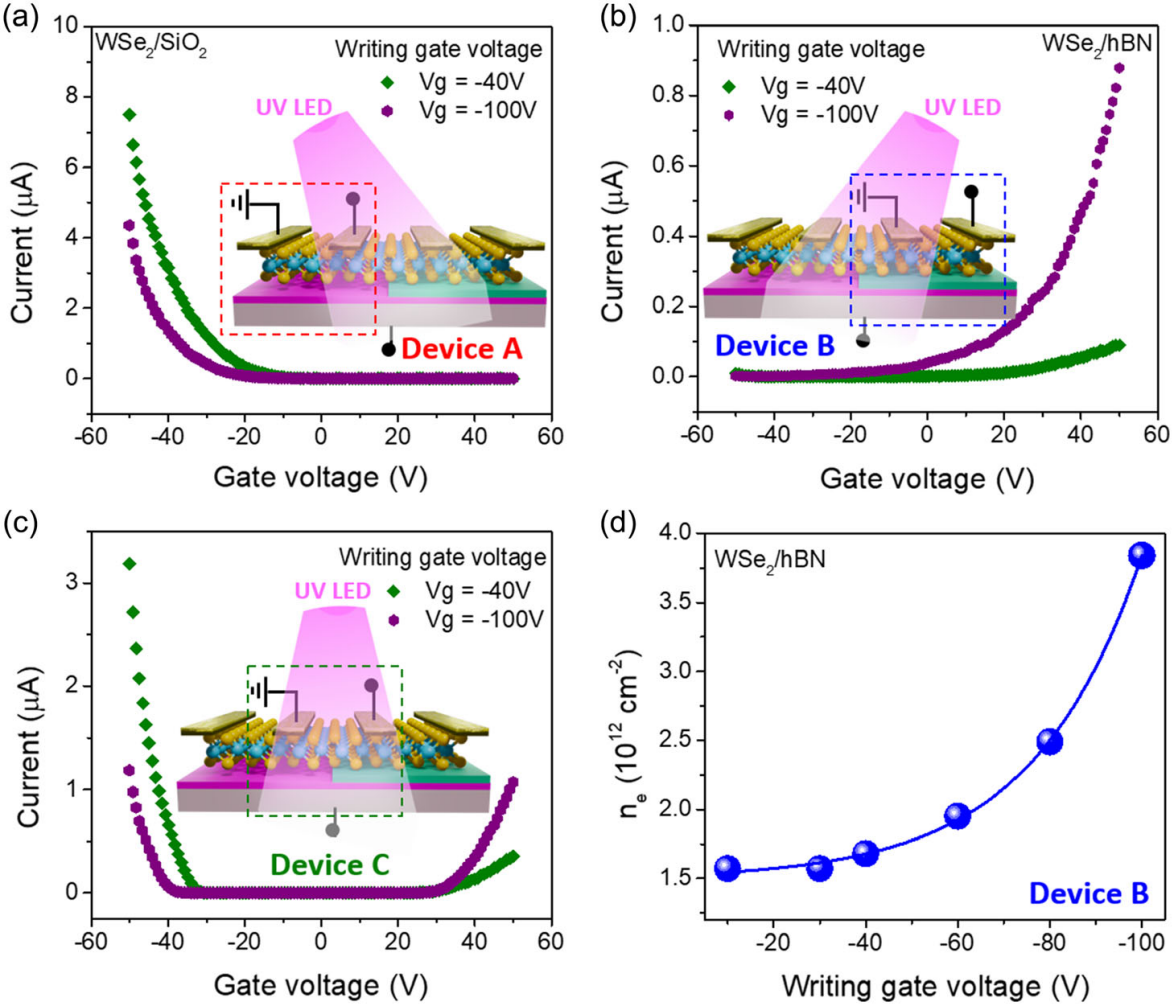
Transport characteristics of various WSe_2_ FETs after photoinduced doping. a) Transfer curves of device A, b) device B, and c) device C after UV writing at different writing voltages (−40 and −100 V) with 5 min of UV illumination. d) Carrier concentrations of device B as a function of writing voltages.

The fabricated devices were then exposed to a UV LED (*λ* 280 nm, power 8 mW cm^−2^) under a constant *V*_gs_ for 5 min (referred to as writing voltage) and then the transfer characteristics of device A, device B, and device C (one electrode on WSe_2_/hBN and another electrode on WSe_2_/SiO_2_ thereby creating a device at the junction of hBN) were measured. **Figure** **2** shows the transfer curves of the three device types after being written with different voltages (−40 and −100 V). The transfer curves demonstrate that tunable n‐type doping can be achieved in device B and that the polarity of the device can be transformed from ambipolar to unipolar n‐type for larger writing voltages (Figure 2b). On the contrary, device A shows a relatively weak response to negative writing voltages and a small decrease in hole current is observed. The n‐type doping in device A is probably a result of Se vacancies formation in WSe_2_ under UV illumination,^[^ ^30^ ^]^ while the polarity of the device remained the same (unipolar p‐type) (Figure 2a). The output curves of device A and device B at writing voltage of –100 V are presented in Figure S5, Supporting Information. Furthermore, repeatable ambipolar transport was observed in device C (Figure 2c) when the writing voltage was set to –100 V under UV exposure. The output characteristics of device C after writing (at –100 V) show a gate dependent rectifying effect (Figure S6, Supporting Information) confirming the formation of a lateral WSe_2_ p–n homojunction diode. From the transfer curves (Figure 2b), it was also observed that as the writing voltage becomes more negative, the on current increases (electron branch) and the threshold voltage decreases, leading to an increase in electron doping in the WSe_2_ channel.^[^ ^38^ ^]^ The electron carrier concentration as a function of writing voltage in WSe_2_/hBN channel was calculated using the parallel‐plate capacitor model^[^ ^39^ ^]^ (Equation (1))

(1) $$n_{e}=C_{t}\left(V_{g}–V_{\text{th}}\right)/e$$

where *C*_t_ is the total capacitance of the SiO_2_ and hBN substrate layers, *V*_g_ is the gate voltage, *V*_th_ is the threshold voltage, and *e* is the electron charge. The electron carrier concentration for device B is estimated as 1.68 × 10^12^ cm^−2^ for a writing voltage of –40 V and increases to 3.84 × 10^12^ cm^−2^ as writing voltage changes to −100 V (Figure 2d). Furthermore, the electron mobility of device B follows a similar trend. On the contrary, device A exhibits the opposite behavior wherein by increasing the absolute value of the writing voltage, threshold voltage increases, and on current decreases (hole branch), suggesting n‐type doping. The estimated hole concentration decreases from 2.86 × 10^12^ to 2.58 × 10^12^ cm^−2^ when writing voltage changes from −10 to −100 V for device A (Figure S7, Supporting Information).

### 2.3 Material Characterization After Writing

**Figure 3:**
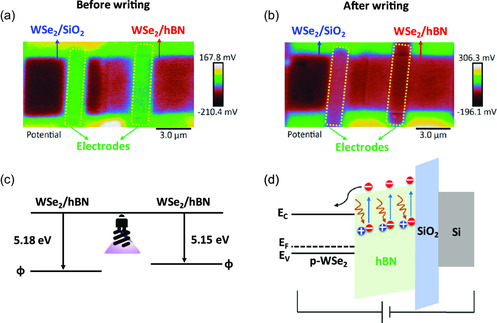
Surface potential and band diagram. a,b) AM‐KPFM images of WSe_2_/SiO_2_ and WSe_2_/hBN FETs before and after writing, c) work function extracted from AM‐KPFM data, and d) schematic illustrating photoinduced‐doping mechanism in WSe_2_/hBN heterostructure under UV light illumination and negative gate voltage.

To further investigate the effect of the photoinduced doping on the WSe_2_ devices, amplitude modulated Kelvin probe force microscopy (AM‐ KPFM) was used to gain an insight into the surface potential and by extension work function of the devices A‐C before and after UV writing. In this case, the device was written for 5 min with a writing voltage of −50 V. Experimental details and topography details are shown in the experimental section and Figure S8, Supporting Information. As shown in **Figure** **3**a, the AM‐KPFM scans show that in its native state, device A has a surface potential of around 60 mV less than device B. This is further evidence that hBN reduces the Fermi level pinning and thereby reduces the work function of WSe_2_ on hBN (device B). After the writing process (Figure 3b), the difference in the surface potential changes to around 100 mV. This difference in surface potential is likely an underestimate of the true difference due to the averaging nature of AM‐KPFM.^[^ ^40^ ^]^ Previous works^[^ ^41^ ^]^ have shown that WSe_2_ lateral homojunctions can be created using ion‐beam irradiation where the area of the sample which has been irradiated completely loses its p‐type conductivity. Their KPFM analysis showed that there was a difference of 55 mV between the irradiated and pristine areas of the WSe_2_ flake, sufficient enough to turn off conductivity in the irradiated sections of the flake. More details are given in Supporting Information Figure S9, Supporting Information. The change in surface potential can be translated to a change in work function after calibration of the tip (details in the experimental section) as shown in Figure 3c. The photoinduced‐electron doping mechanism in WSe_2_ on hBN is presented in the band diagram shown in Figure 3d wherein by illuminating UV light, electrons in the defect states of hBN acquire enough energy to be excited to the conduction band of hBN^[^ ^42^ ^]^ and due to applied negative gate voltage these excited electrons move toward the conduction band of WSe_2,_ leading to n‐type doping in WSe_2_. The positively charged defects remain in hBN and alter the local electric field.^[^ ^43^, ^44^ ^]^ The electron transfer process from hBN to WSe_2_ persists until the applied writing voltage is completely neutralized by the positively charged defects.^[^ ^42^ ^]^

**Figure 4:**
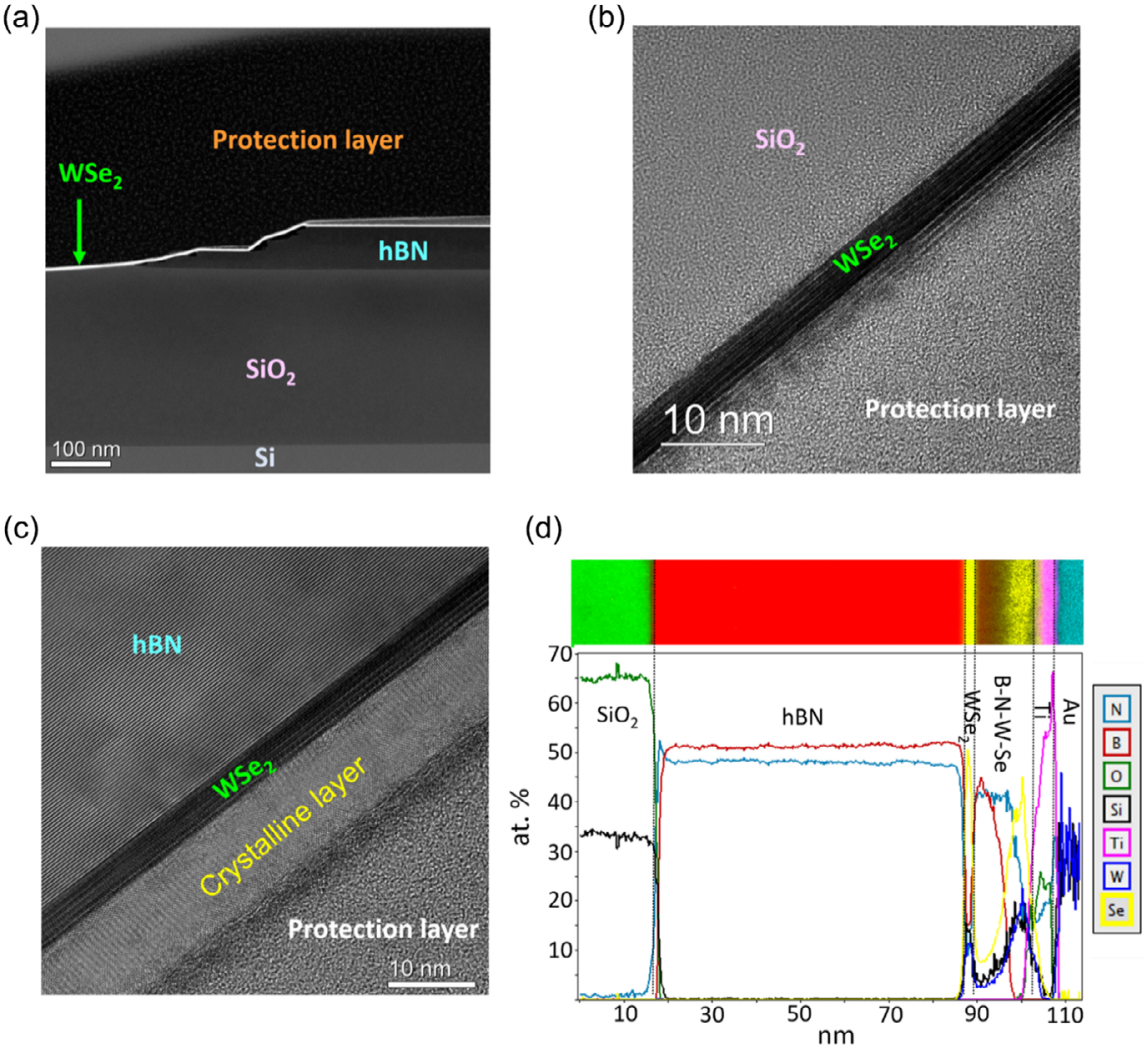
HRTEM characterization. a) After writing, high angle annular dark field (HAADF) STEM image of WSe_2_/SiO_2_ and WSe_2_/hBN region of the device, b,c) high resolution bright field TEM images of WSe_2_/SiO_2_ and WSe_2_/hBN, and d) quantified profile (based on EELS mapping) of the chemical composition below the electrode in device B. In EELS, the Si K‐ and the W *M*‐edges overlap and therefore give artificial Si contents in the crystalline layer on top of WS_2_.

The effect of the photoinduced doping observed in our devices was investigated using HRTEM on the device shown in Figure S10, Supporting Information, after it had been written with a writing voltage of ‐ 100V. A focused ion beam/scanning electron microscope (FIB/SEM) was used to create a thin lamella, the details of which are presented in the experimental section and the supplementary information. **Figure** **4**a shows a high angle annular dark field STEM image of the device, centered around the edge of the hBN flake (device C). As observed, the edge of the hBN flake is not sharp and there is a small incline over a length of about 200 nm with small areas where the WSe_2_ flake is not in contact with the substrate. These areas or “air‐gaps” create small regions of reduced doping and low parasitic capacitance which can lead to an increased conductivity of device C as previously demonstrated.^[^ ^16^ ^]^ Further details of the “air‐gaps” are shown in Figure S11, Supporting Information. Figure 4b,c show high resolution bright field TEM images of devices A and B. We observe an unknown crystalline layer on the surface of WSe_2_ which is on hBN. This layer is absent on the WSe_2_ which is on SiO_2_. In Figure 4d, a line profile of the chemical composition, based on electron energy loss spectroscopy (EELS) mapping, is shown from a region under the electrode of device B. The unknown crystalline layer is composed of a mixture of B, N, W, and Se. The first part of this layer is rich in B and N. Firstly, the B concentration starts to decay, then the N concentration. The top of the crystalline layer is rich in W and Se. This additional layer is observed both under and on the sides of the Au contacts on top of the WSe_2_ on hBN (Figure S12 and S13, Supporting Information). We propose that this layer, which contributes to the doping of the WSe_2_ in addition to the defect states in the hBN substrate, originates from diffusion processes which may occur during the writing process that occurs at high electrical fields. It is well known that this type of gate stress test can lead to instabilities in FETs.^[^ ^45^ ^]^ Additionally, transfer curves of device C after UV writing at different writing voltages (Figure S14, Supporting Information) suggest the involvement of two mechanisms: one at low writing voltages (−10 to −30 V) and one at greater writing voltages. One hypothesis is that at small writing voltages, only Se vacancies and interface traps contribute to the doping process however at larger writing voltages, diffusion processes transport B and N to the surface as seen in Figure 4.

### 2.4 WSe_2_ Logic Inverter Demonstration

**Figure 5:**
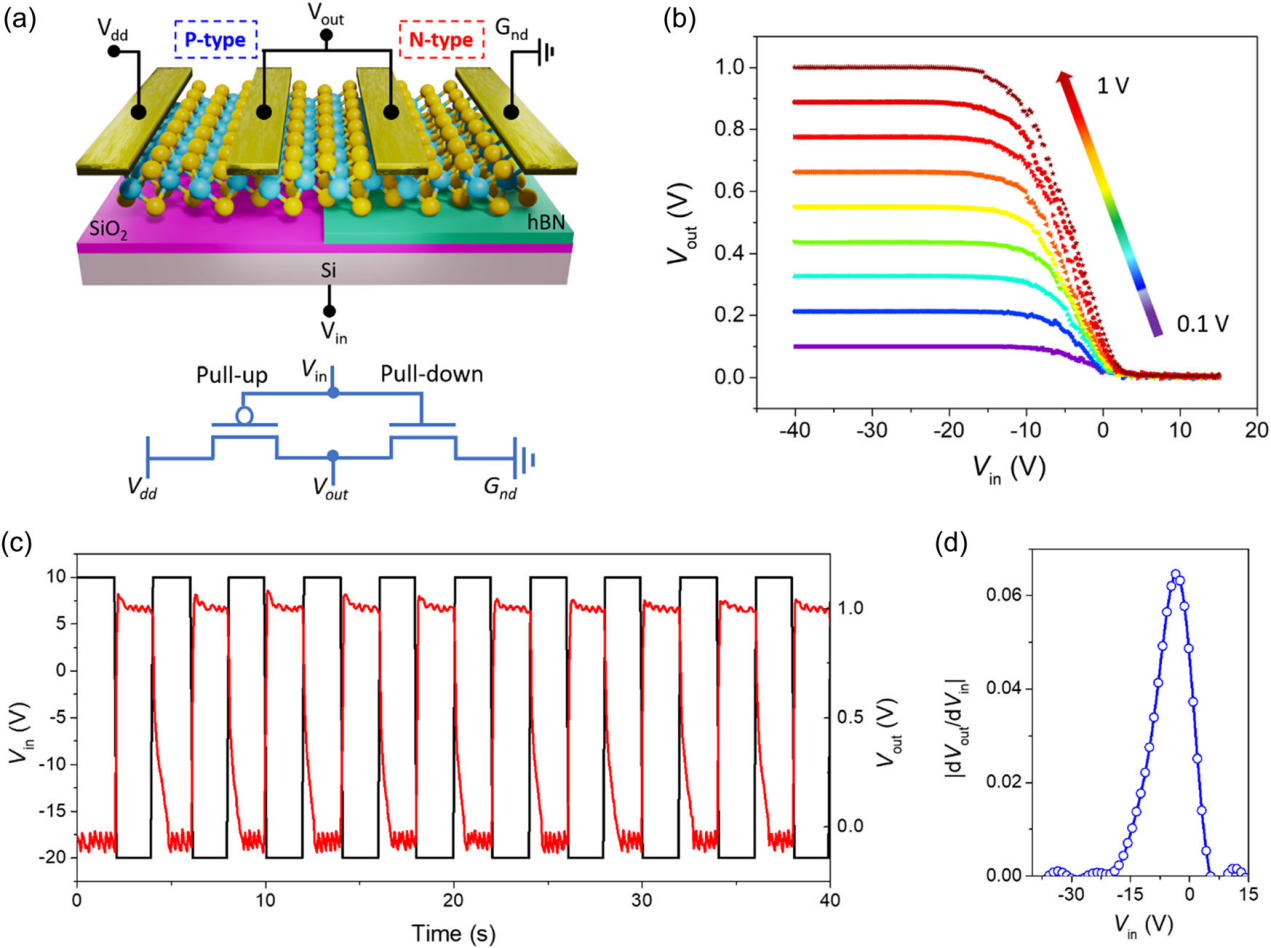
WSe_2_ logic inverter. a) WSe_2_ inverter schematic and circuit diagram, b) voltage transfer characteristics of WSe_2_ inverter at different supply voltages, c) practical demonstration of inverting circuit, where the input signal is inverted, and d) voltage gain of the WSe_2_ inverter at supply voltage of 1 V.

As a proof of concept of our tunable n‐type doping mechanism, we demonstrate a complementary inverter using the same device as shown in Figure 1a. The photoinduced doping along with electrostatic activation allowed us to create unipolar n‐type transport in the WSe_2_/hBN channel while keeping the WSe_2_/SiO_2_ channel unipolar p‐type. To form a complementary inverter, the WSe_2_/SiO_2_ transistor (pull‐up) was externally connected in a series with the WSe_2_/hBN transistor (pull‐down). The input signal (*V*_in_) was applied to the common back gate and the output signal was collected from the shorted source drain terminal of pull‐up and pull‐down transistor respectively as shown in **Figure** **5**a. Figure 5b shows the transfer curves of the WSe_2_ complementary inverter at different supply voltages (*V*_dd_ = 0.1–1 V). When a negative gate voltage is applied to the inverter, the WSe_2_/SiO_2_ transistor (device A) is turned ON while the WSe_2_/hBN transistor (device B) is at OFF state, resulting in the supply voltage of pull‐up transistor appearing at the output terminal which represents the logic “1”. In contrast, when a positive gate voltage is applied to the inverter's input, the WSe_2_/SiO_2_ transistor (device A) is turned OFF, therefore, no supply voltage appears at the output terminal and thus represents the logic “0” state (Figure 5b). Figure 5c clearly indicates that the input square wave signal is inverted with a finite delay and rise time due to a large subthreshold swing.^[^ ^46^ ^]^ Figure 5d illustrates the voltage gain of the inverter, which is an important performance parameter and is expressed as *V*_gain_ = |*dV*_out_/*dV*_in_|. The observed modest voltage gain well below the threshold of 1 for practical applications is directly related to the low capacitance of our device, originating in the 300 nm thick SiO_2_ layer. Replacing this layer with a thinner layer of SiO_2_ or preferably a high‐k gate dielectric, will significantly increase voltage gain, allow better switching control and improve overall performance of the inverter.^[^ ^46^ ^]^ Power consumption is also an important parameter to evaluate the performance of the logic inverter. Our fabricated inverter demonstrates a peak power consumption of 2.25 nW. The calculated power consumption is illustrated in Figure S15, Supporting Information, which – a consequence of the low voltage gain – favourably aligns with values reported in the literature, as summarized in **Table** **1**. To study the stability of photo‐induced doping, the performance of the fabricated inverter was continuously monitored for 4 weeks in the dark environment, and negligible degradation was observed (Figure S16, Supporting Information). More complexed logic circuits are possible by pre‐patterning the hBN substrate prior to WSe_2_ transfer.

**Table 1:** Comparison of performance parameters of reported 2D semiconductor‐based inverters

Materials	Doping method	Bias voltage [V]	Gain	Power consumption [nW]	Refs.
MoTe_2_ p‐n homojunction	Laser‐induced doping (532 and 355 nm)	0.1	0.03	–	[30]
p‐MoSe_2_/n‐MoSe_2_	Substitutional doping	10	34	127	[49]
p‐WSe_2_/n‐WS_2_	Intrinsic property	5	1.5	–	[50]
p‐WSe_2_/n‐WSe_2_	Chemical doping (iodine vapor)	3	0.8	–	[51]
MoTe_2_ p‐n homojunction	ALD‐induced doping	2	29	80	[23]
MoTe_2_ p‐n homojunction	Chemical doping	1	–	90	[52]
P‐MoSe_2_/n‐MoSe_2_	Substitutional doping	1	0.5	–	[53]
WSe_2_/SiO_2_–WSe_2_/hBN p‐n homojunction	UV LED‐induced doping	1	0.06	2.25	This work

## 3 Conclusion

In conclusion, we have shown that WSe_2_ field effect transistors on hBN substrate exhibited ambipolar behaviour, allowing both n‐type and p‐type conduction, while the devices on SiO_2_ substrate exhibited unipolar p‐type behaviour. The optical writing induced tuneable n‐type doping in the WSe_2_ devices on hBN substrate, leading to a change in carrier concentration, carrier type and mobility. The study also revealed the formation of a crystalline layer containing boron, nitrogen, tungsten, and selenium on the surface of WSe_2_, suggesting a diffusion‐based doping mechanism during the writing process. Finally, an optical writing strategy was used to demonstrate a complementary logic inverter based on homogenous WSe_2_ transistors.

## 4 Experimental Section

4.1

4.1.1

##### Device Fabrication

Few‐layer WSe_2_ were mechanically exfoliated and transferred on a pre‐prepared 60 nm thick hBN flake and SiO_2_ substrate by a dry transfer technique in such a way that the WSe_2_ flake lies on both hBN and SiO_2_ (Figure S1, Supporting Information). E‐beam lithography (EBL) and reactive ion etching was used to pattern the WSe_2_ channel. Finally, EBL and electron‐beam evaporation were used to realize the electrodes followed by lift‐off of titanium/gold (5/110 nm) metals.

##### Electrical Characterization

Electrical characterization of the devices was performed using a two‐point configuration with a Keithley 2440 source meter. The gate voltage was applied by a Keithley 2450 instrument, and all data were recorded by a customized *LabVIEW* program. The field‐effect carrier mobility (*μ*) was calculated by using Equation (2).

(2) $$\mu =\left[dI_{\text{ds}}\text{/}dV_{g}\right]\;\times \;\left[L/\left(WC_{t}V_{\text{ds}}\right)\right]$$

where *dI*_ds_/*dV*_g_ is the transconductance and obtained from the transfer curve, *L* and *W* are the length and width of the channel, respectively, *C*_t_ is the total capacitance per unit area and *V*_ds_ is the drain‐source voltage.

##### AM‐KPFM Characterization

AM‐KPFM was carried out using a Bruker Multimode AFM. A Pt/Ir conductive tip (SCM‐PIT‐V2) was used for the KPFM experiments. Here, the contact potential difference (VCPD) between the film and tip is compensated by applying an external bias voltage. The work function of the sample (*ϕs*) can be obtained from the VCPD via the following Equation (3)^[^ ^47^, ^48^ ^]^

(3) $$\text{VCPD}=\frac{\left(\phi M-\phi s\right)}{q}$$

where *ϕM* is the work function of the metal tip and *q* is the elementary charge.

##### TEM Characterization

TEM sample preparation was performed using a Helios G4 UX dual‐beam focused ion beam (FIB). A 3 μm thick carbon layer was deposited on the region of interest prior to cutting out the TEM lamella. The first part of this carbon protection layer was deposited by e‐beam assisted deposition to avoid Ga^+^ damage in the region of interest. During ion beam thinning, an ion‐beam acceleration voltage of 30 kV was used for coarse thinning with final thinning performed at 5 and 2 kV on either side of the lamellae to minimize surface damage. TEM was performed at 200 kV using a double Cs aberration corrected cold FEG JEOL ARM 200FC. Spectroscopy was performed in scanning transmission electron microscopy (STEM) mode by simultaneous acquisition of energy dispersive spectroscopy (EDS) and electron energy loss spectroscopy (EELS) data. EDS was done with a 100 mm^2^ Centurio detector covering a solid angle of 0.98 sr, while EELS were performed with a GIF Quantum ER operating in dual‐EELS mode.

## Conflict of Interest

The authors declare no conflict of interest.

## Supporting information

Supplementary Material

## Data Availability

The data that support the findings of this study are available from the corresponding author upon reasonable request.
