# Supplementary material for: Two‐Dimensional Heterostructure Complementary Logic Enabled by Optical Writing
Source: Small Sci. 2024 Mar 1;4(5):2300319. doi: 10.1002/smsc.202300319 (PMC11935277; doi:10.1002/smsc.202300319)
Supplement: Supplementary file 1 — Supplementary Material [file SMSC-4-2300319-s001.pdf]

Supporting information

Two-dimensional Heterostructure Complementary Logic Enabled by Optical Writing

*Ayaz Ali, Matthias Schrade, Wen Xing, Per Erik Vullum, Ozhan Koybasi, Takashi Taniguchi, Kenji Watanabe, and Branson D. Belle**

S1. Heterostructure assembly:

The process for 2D heterostructure assembly of WSe_2/hBN is schematically illustrated in **Figure S1**. WSe_2 flake was mechanically exfoliated onto PMMA/PVA coated silicon substrates (substrate 1) as shown in Figure S1 a. Then WSe_2/PMMA stack was separated from the substrate 1 by dissolving a sacrificial polymer layer (PVA) between the PMMA and Si surface. The hBN fake was mechanically exfoliated on Si/SiO_2 substrate (substrate 2) as shown in figure S1 b. In the last, WSe_2/PMMA stack was then flipped, aligned and transferred on the top of hBN flake under an optical microscope as shown in Figure S1 c. Finally, the PMMA support layer was dissolved in acetone.

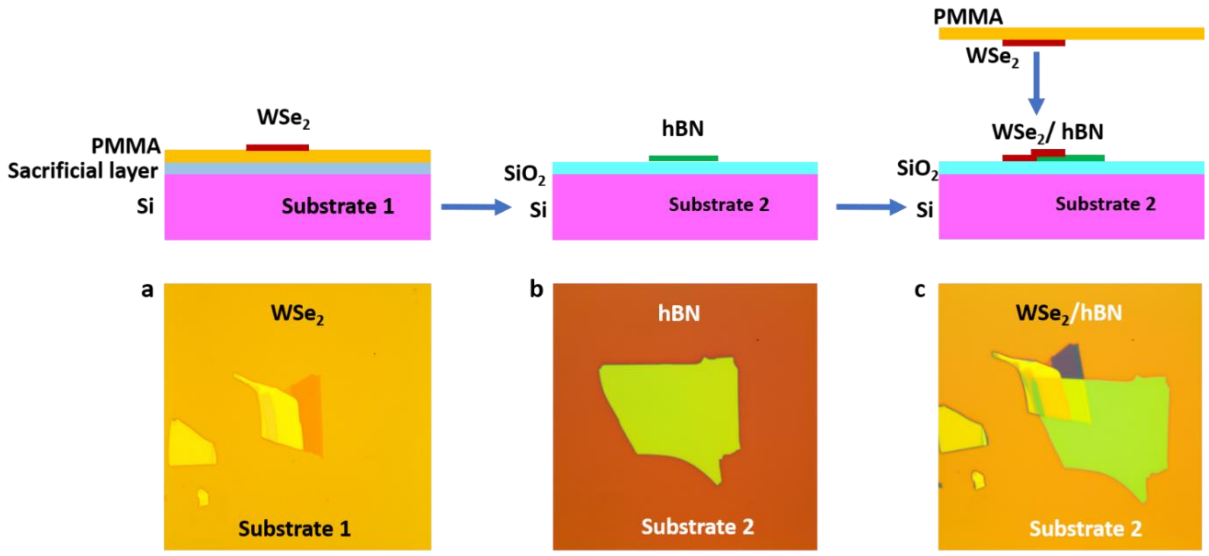

Figure S1. WSe_2/hBN Heterostructure assembly process.

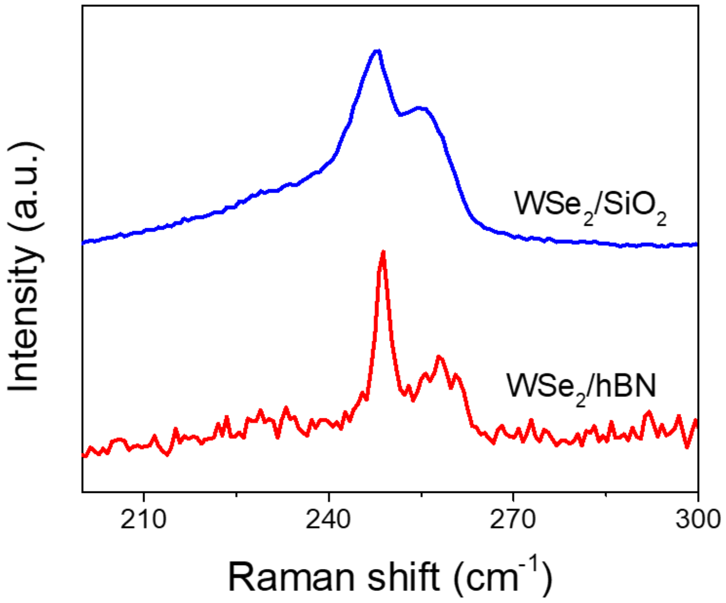

Figure S2. Raman spectrum of WSe_2 on SiO_2 and WSe_2 on hBN substrate

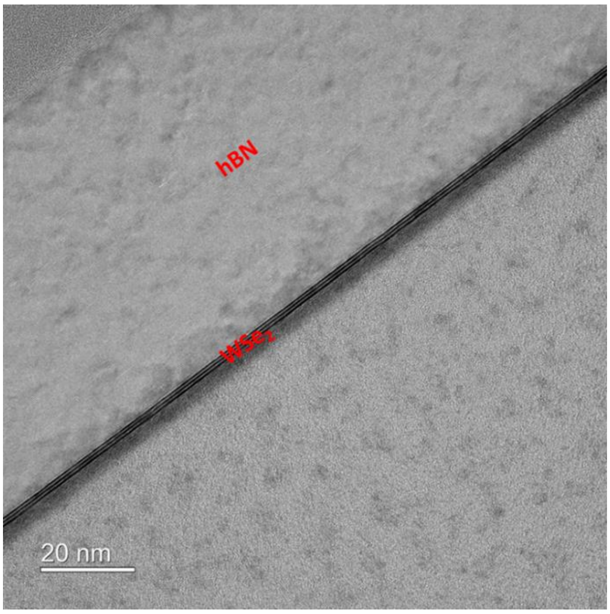

Figure S3. Bright field TEM image of WSe_2/hBN region of a pristine device (before photoinduced doping process).

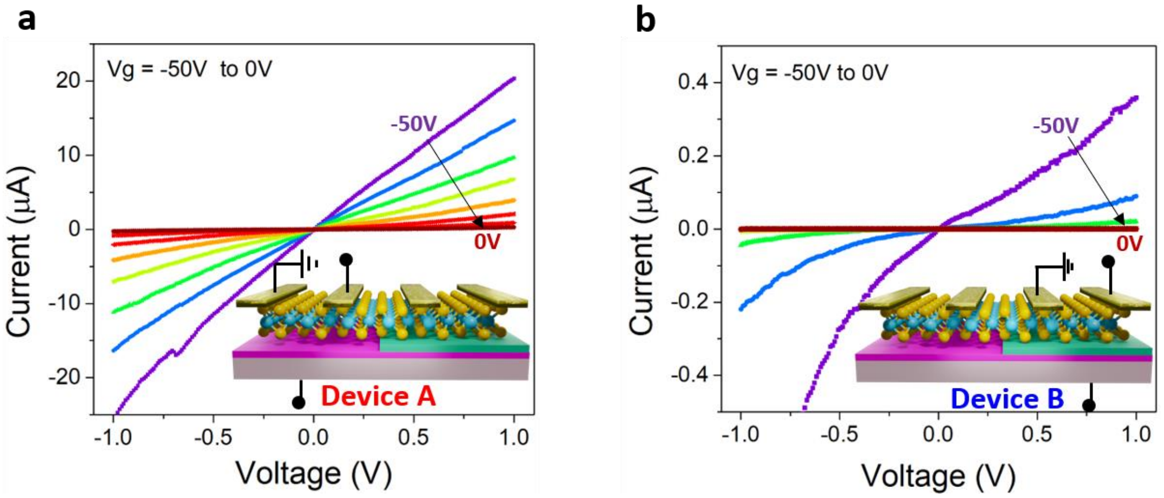

Figure S4. a) Output characteristics of as fabricated pristine WSe_2/SiO_2 and b) WSe2/hBN FETs under different gate bias (Vg = -50V to 0V).

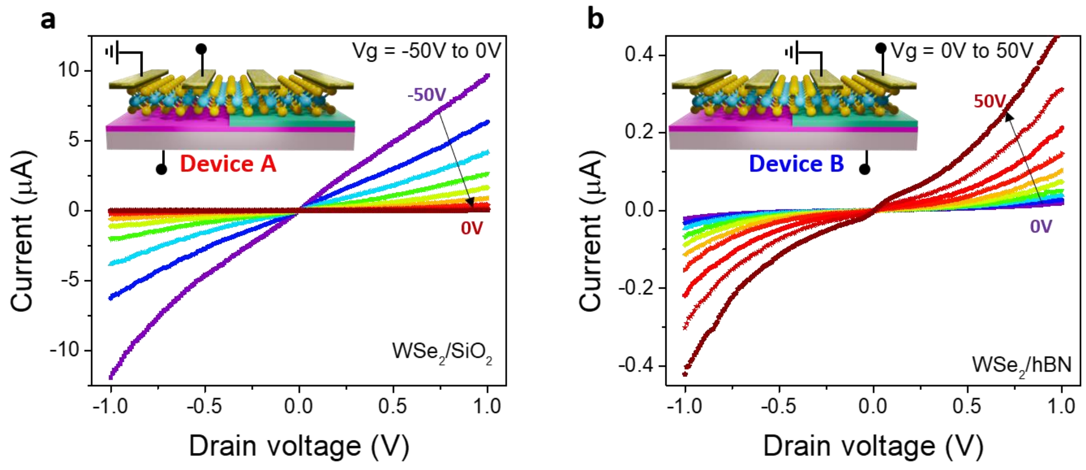

Figure S5. a) Output characteristics of WSe_2/SiO_2 and b) WSe2/hBN FETs under different gate bias recorded after UV writing at –100 V Vg.

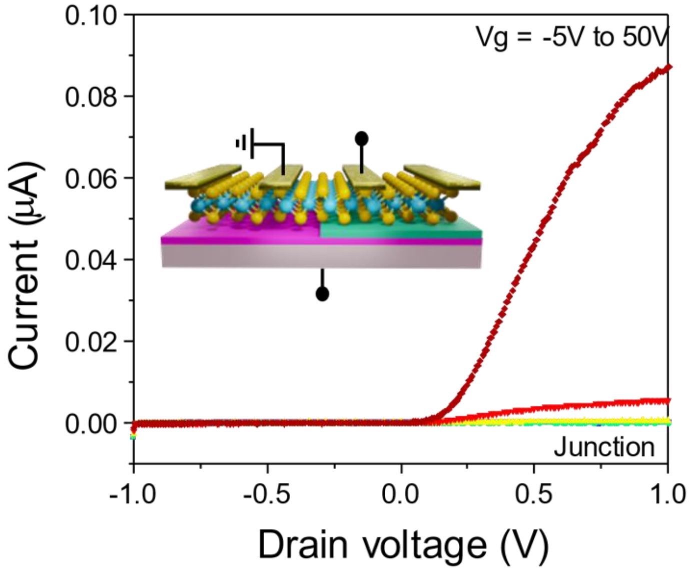

Figure S6. After writing at - 100 V, output characteristics of junction device (device C) under different gate bias (-5V to 50 V).

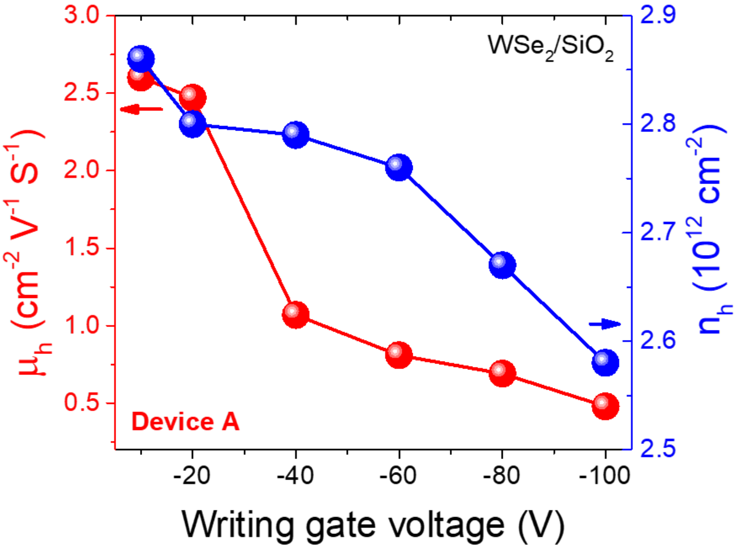

Figure S7. Carrier concentrations and hole mobility of device A (WSe_2/SiO_2) as a function of writing voltages

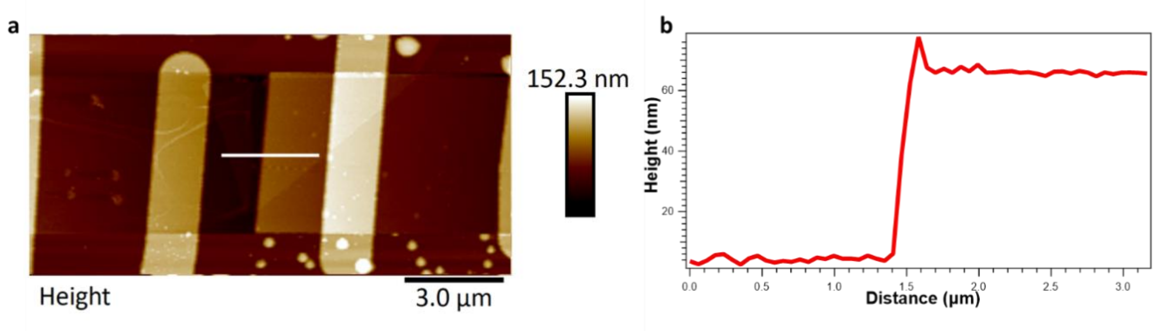

Figure S8. a) AFM topography image of devices A -C b) line trace showing hBN thickness

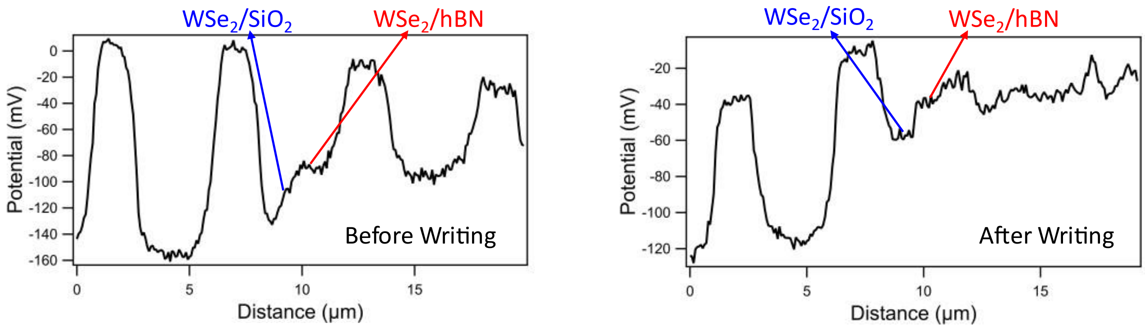

Figure S9. Expanded view line profile of the surface potential of the device before and after UV writing

It should be noted that the surface potential of WSe_2 on SiO_2 of Device C does not fully align with that of Device A due band bending which occurs at the junction of SiO_2 and hBN both before and after writing. After writing the potential of the WSe_2 on SiO_2 of Device C is pulled up even further towards the potential of WSe_2 of hBN of Device C due to band bending after writing.

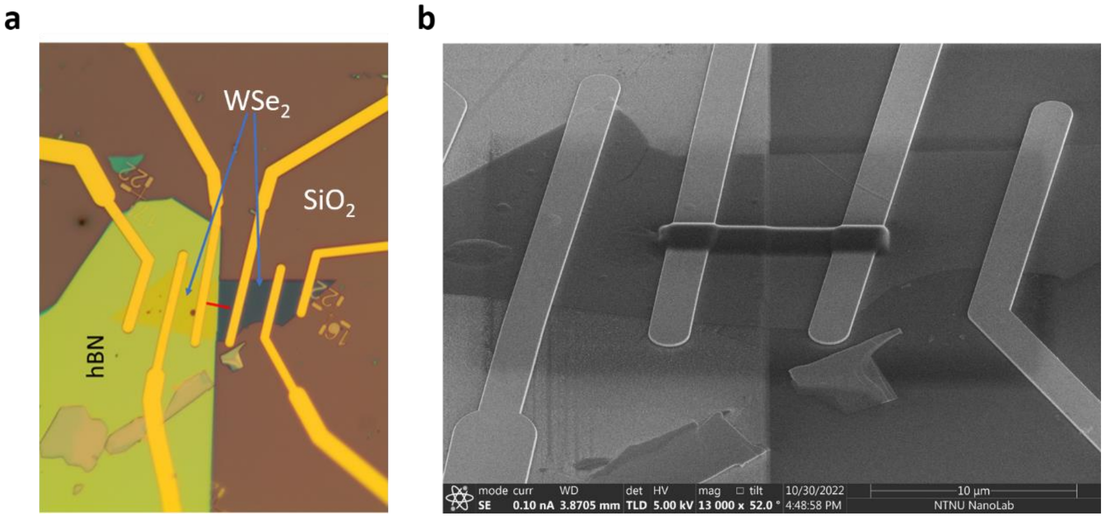

Figure S10. Optical and SEM images of the WSe_2 device.

Optical and SEM images of the WSe_2 device that was used to study the effect of photoinduced doping on structural changes. In the SEM image, a thin carbon protection layer is deposited by e-beam assisted deposition and shows the location of the TEM lamella

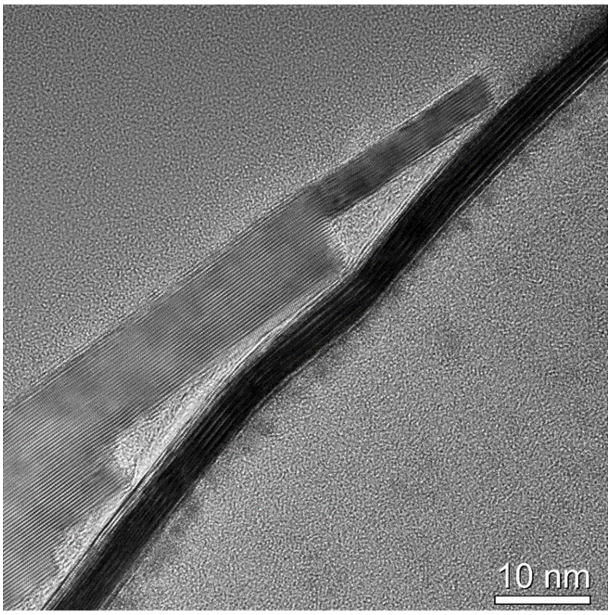

Figure S11. Bright field TEM image of the 'air-gaps' created due to terraces at the edge of the hBN substrate.

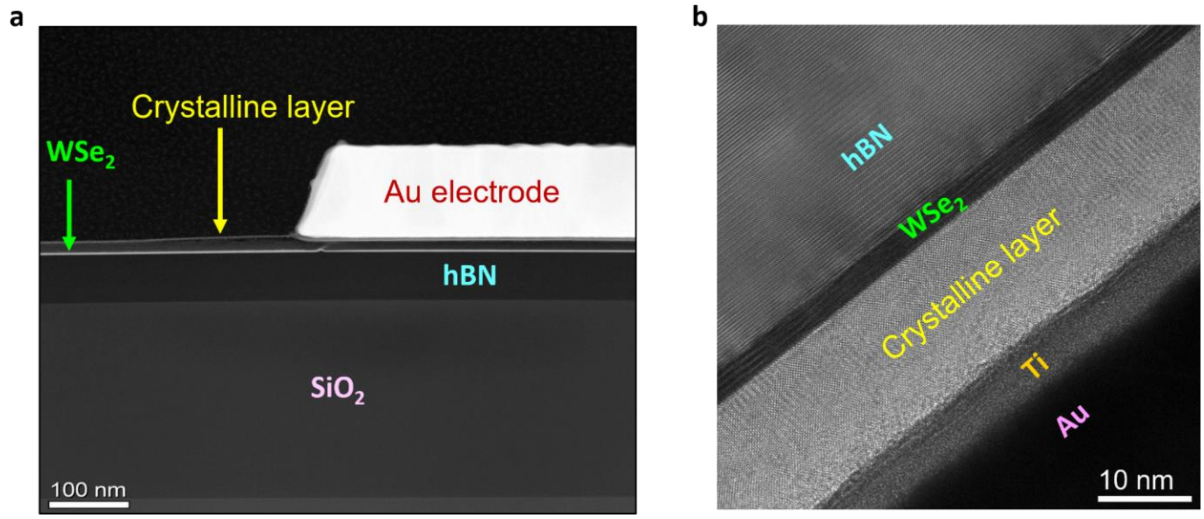

Figure 12. After writing, high angle annular dark field (HAADF) STEM image and high-resolution bright field TEM image of the WSe2/hBN device under the electrode region.

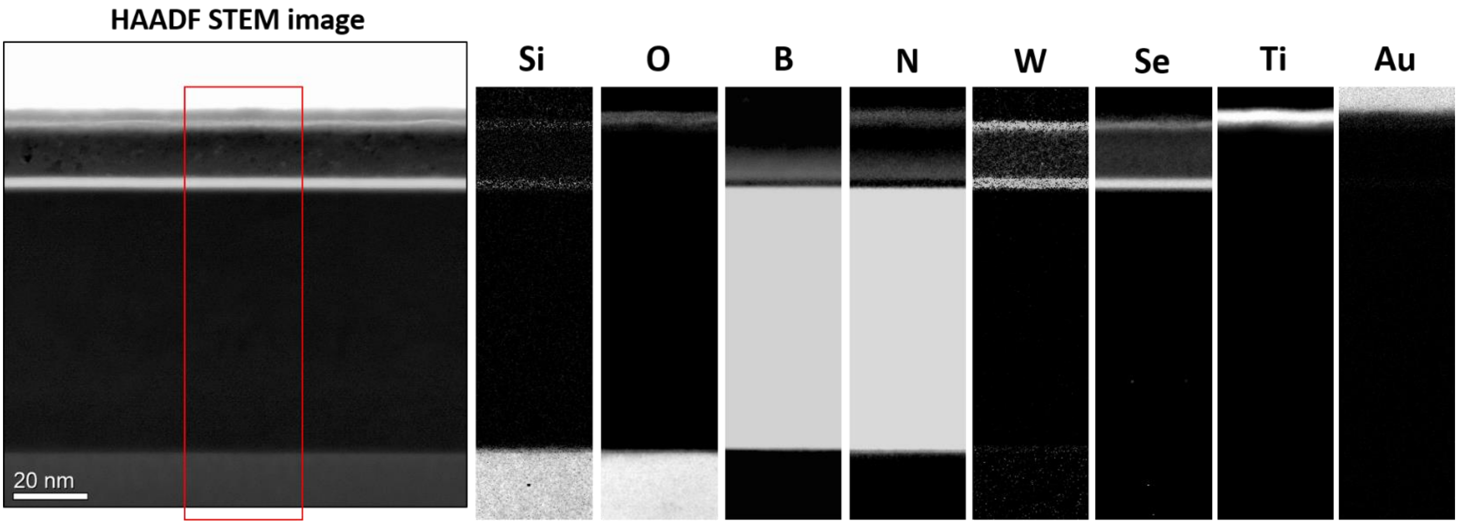

Figure S13. HAADF STEM image and Element mapping by simultaneous EDS/EELS. All element maps are taken from the EELS data except the Au map which is generated from the EDS data. In EELS, the Si K- and W M-edges overlap. This overlap gives an artificial Si signal wherever W is present.

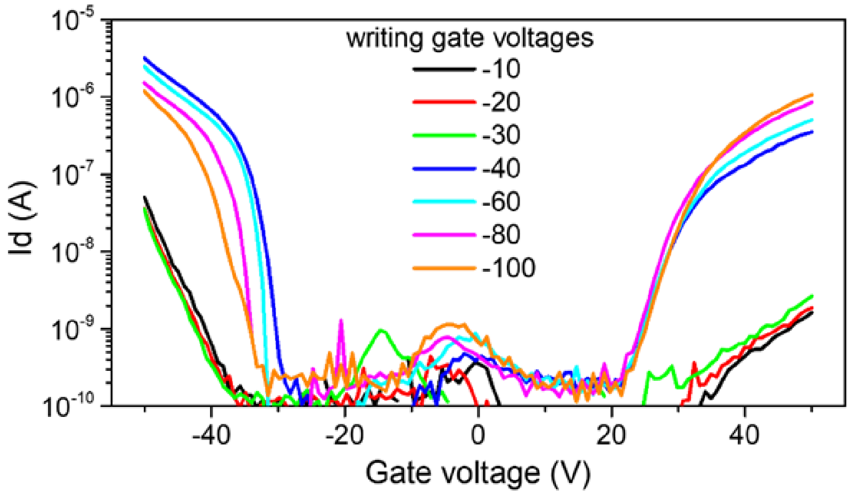

Figure S14. Transfer curves of device C after UV writing at different writing voltages (-10V to –100V) with 5 minutes of UV illumination.

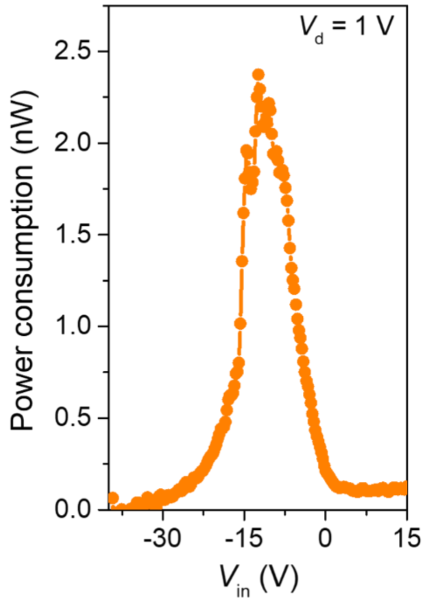

Figure S15. Power consumption of fabricated WSe_2 homojunction inverter at V_{ds} of 1 V. The power consumption (P) of the WSe_2 homojunction inverter was calculated using P = Id x V_d, where I_d is the drain current and V_d is the drain-source voltage.

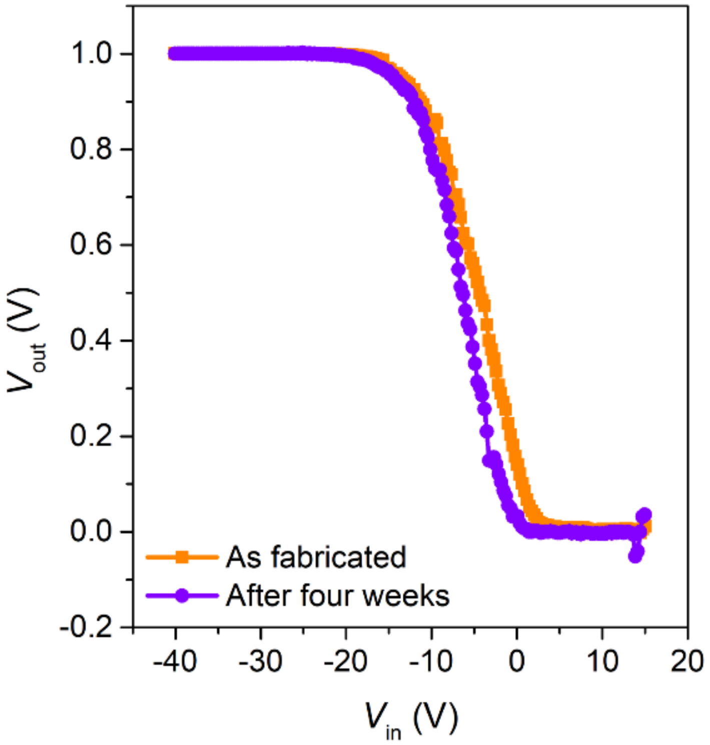

Figure S16. Voltage transfer characteristics of fabricated WSe_2 homojunction inverter at supply voltages of 1 V and measured after 4 weeks in dark environment.
